# Plant circadian rhythms regulate the effectiveness of a glyphosate-based herbicide

**DOI:** 10.1038/s41467-019-11709-5

**Published:** 2019-08-16

**Authors:** Fiona E. Belbin, Gavin J. Hall, Amelia B. Jackson, Florence E. Schanschieff, George Archibald, Carl Formstone, Antony N. Dodd

**Affiliations:** 1 School of Biological Sciences, University of Bristol, Bristol, BS8 1TQ UK; 2 Syngenta, Jealott’s Hill International Research Centre, Warfield, Bracknell, RG42 6EY UK

**Keywords:** Circadian rhythms in plants, Light responses

## Abstract

Herbicides increase crop yields by allowing weed control and harvest management. Glyphosate is the most widely-used herbicide active ingredient, with $11 billion spent annually on glyphosate-containing products applied to >350 million hectares worldwide, using about 8.6 billion kg of glyphosate. The herbicidal effectiveness of glyphosate can depend upon the time of day of spraying. Here, we show that the plant circadian clock regulates the effectiveness of glyphosate. We identify a daily and circadian rhythm in the inhibition of plant development by glyphosate, due to interaction between glyphosate activity, the circadian oscillator and potentially auxin signalling. We identify that the circadian clock controls the timing and extent of glyphosate-induced plant cell death. Furthermore, the clock controls a rhythm in the minimum effective dose of glyphosate. We propose the concept of agricultural chronotherapy, similar in principle to chronotherapy in medical practice. Our findings provide a platform to refine agrochemical use and development, conferring future economic and environmental benefits.

## Abstract (web summary)

Glyphosate is the world’s most widely-used herbicide. Here, Belbin et al. show that plant responses to glyphosate, and therefore herbicide activity, depend on plant circadian rhythms suggesting that considering the time-of-day of application could lead to more efficient agrochemical use.

## Introduction

Global food requirements demand crop production increases of 100–110% by 2050^1^. Weeds cause estimated yield losses of 34%^2^, with herbicides being a tool to tackle these losses and also enhance harvest management^3^. Glyphosate (*N*-(phosphonomethyl) glycine) is the most widely used herbicide active ingredient, with $5 billion and $11 billion spent annually on glyphosate-containing products in the USA and worldwide, respectively^4^. Glyphosate-based formulations are used on >350 million hectares worldwide, involving about 8.6 billion kg of glyphosate annually^4^. This scale of glyphosate use makes strategies to enhance its utility commercially and environmentally attractive.

Intriguingly, the herbicidal effectiveness of glyphosate can depend upon the time of day of application^5–8^. One mechanism that influences the timing of responses of plants to their environment is the circadian clock. Circadian rhythms are biological cycles with a period of about 24 h that persist in the absence of external cues. In plants, circadian rhythms are generated by a series of interlocked transcription-translation loops and posttranslational mechanisms that are known collectively as the circadian oscillator. The phase of the circadian oscillator is adjusted to match the phase of the environment through the process of entrainment in response to light, temperature and metabolic cues, and the circadian oscillator regulates metabolism, development and physiology primarily through regulation of gene expression. The circadian oscillator also modulates the responses of plants to a variety of environmental cues so that the response depends on the time of day, through a process known as circadian gating.

We hypothesised that circadian regulation might underlie certain rhythmic responses of plants to glyphosate because the circadian clock co-ordinates the timing of many physiological and developmental processes in plants^9,10^. In Arabidopsis, 6–15%^9,11^ of the transcriptome is circadian-regulated with up to 89%^12^ having the potential for rhythmic behaviour under a range of environments. The resulting rhythms of metabolism and development present a variety of potential rhythmic targets for agrochemicals. This is reminiscent of over half of the 100 highest grossing prescribed drugs in the USA having circadian-regulated targets in the mouse^13^, providing a basis for temporal variation in drug sensitivity that underpins chronotherapy. We reasoned that pervasive circadian regulation in plants might underlie rhythmic responses to certain chemical applications, and tested this notion for glyphosate-based herbicides due to their widespread use. This is an important question, because rhythmic responses of plants to agrochemicals introduce novel opportunities to refine or reduce agrochemical use.

## Results

### A subset of glyphosate-responsive transcripts are rhythmic

We wished to establish why the effectiveness of glyphosate (*N*-(phosphonomethyl)glycine) can depend on the time of day of application^5–8^. Glyphosate inhibits 5-enolpyruvyl-shikimate-3-phosphate synthase (EPSPS) within the shikimate pathway^14^, eventually killing the plant (Supplementary Fig. 1). We identified cellular processes influenced by both circadian rhythms and glyphosate, by interrogating transcriptome data from the experimental model *Arabidopsis thaliana*. A subset of glyphosate-responsive transcripts^15^ oscillate under light-dark cycles (Supplementary Fig. 2A)^16,17^ or are circadian-regulated (Supplementary Fig. 2B)^11,18,19^. From these, we identified glyphosate-responsive transcripts that are consistently diel- and circadian-regulated (Supplementary Fig. 2C). This identified 18 and 57 rhythmic transcripts that are glyphosate-induced and repressed, respectively (Supplementary Fig. 2C; Supplementary Data 1). Seventy two percent of glyphosate-induced rhythmic transcripts reach peak abundance at dawn (Supplementary Fig. 2D), whereas rhythmic glyphosate-repressed transcripts oscillate with a range of phases (Supplementary Fig. 2D).

A gene ontology (GO)-term analysis of the 137 transcripts that are glyphosate- and circadian-regulated identified a significant enrichment of this transcript set with an auxin transport GO term (GO:0060918; *P* = 0.024; Benjamini Hochberg correction), whereas the 538 transcripts that are glyphosate- and light/dark regulated is not enriched significantly for auxin-related GO terms. Examining this further, we identified a statistically significant overlap between the set of 75 transcripts that are consistently glyphosate-responsive and both circadian- or light/dark regulated (Supplementary Fig. 2C) and the 549 unique transcripts present within all of the auxin-related GO terms in the Arabidopsis genome (Supplementary Data 1; *P* = 0.03). These overlaps between glyphosate-responsive and auxin-related transcripts might occur because glyphosate inhibits EPSPS, preventing synthesis of the auxin precursor tryptophan^20^, or because glyphosate can inhibit auxin transport^21^. Auxin signalling is circadian-regulated^19^, so we reasoned that interaction between auxin signalling, glyphosate and circadian rhythms might underlie certain nycthemeral or circadian responses to glyphosate. An informative proxy to study this is seedling hypocotyl elongation, which is regulated by the circadian oscillator and phytohormones including auxin^22^. Therefore, we hypothesised that such rhythms might underlie a rhythmic sensitivity of hypocotyl length to glyphosate.

### Rhythms in the sensitivity of hypocotyl length to glyphosate

**Fig. 1:**
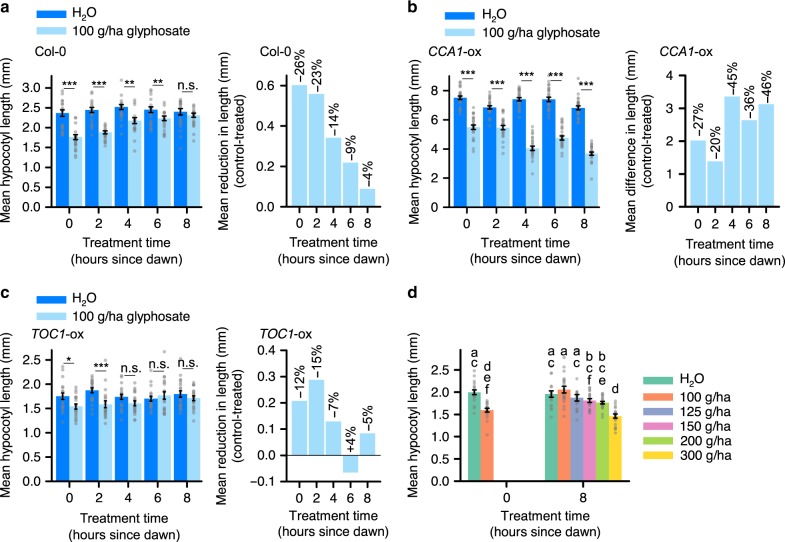
Daily rhythms in glyphosate effectiveness. **a**–**c** Under light/dark cycles, hypocotyls are shorter in seedlings treated with 100 g/ha glyphosate compared with controls at times specified, in **a** Col-0, **b** *CCA1*-ox and **c** *TOC1*-ox seedlings. Graphs show (left) hypocotyl length and (right) change in hypocotyl length caused by glyphosate. **d** Titration of glyphosate concentration required to cause equivalent decrease in hypocotyl length at dawn and dusk in the Col-0 background. **a**–**d** Significance determined by **a**–**c** two-way ANOVA and *t*-tests between control and treatment at each timepoint; **d** one-way ANOVA and Tukey post-hoc analysis. n.s. = not statistically significant, *P ≤ 0.05, **P ≤ 0.01, ***P ≤ 0.001. Data are mean ± s.e.m; *n* = 18–25. Source data are provided in the Source Data file

Glyphosate applied at dawn caused the greatest reduction in hypocotyl length compared with control treatments, whereas hypocotyl length was unaffected by glyphosate applied at dusk (Fig. 1a). This greater sensitivity of hypocotyl length to glyphosate applied at dawn corresponds to a time of elevated auxin signalling^19^. The daily fluctuation in glyphosate sensitivity occurred under 8, 12 and 16 h photoperiods (Fig. 1a, Supplementary Fig. 3 A, B; two-way ANOVA identified a significant interaction between glyphosate treatment and time upon hypocotyl length under 8 h (*P* < 0.001), 12 h (*P* < 0.001) and 16 h (*P* = 0.03) photoperiods, respectively). We performed subsequent experiments under 8 h photoperiods, because the hypocotyls were longer under this photoperiod. During the dark period, hypocotyl length was reduced most by glyphosate applied immediately after dusk and in the pre-dawn period (Supplementary Fig. 3C). The measurements described involved glyphosate application on day 3 after germination. This daily pattern of increased glyphosate sensitivity at dawn occurred also when glyphosate was applied at dawn or dusk on day 5 after germination (Supplementary Fig. 3D). Combined with the predawn increase in glyphosate sensitivity during the dark period (Supplementary Fig. 3C), these data suggest that there are daily cycles of glyphosate sensitivity of hypocotyl length during this period of development, irrespective of any decrease in hypocotyl elongation rate during this period of time.

Circadian arrhythmic *CCA1*-ox^23^ changed the timing of this response, causing a greater reduction of hypocotyl length when glyphosate was applied around dusk (Fig. 1b; significant interaction between treatment and time; *P* < 0.001). Overexpressing the oscillator component TOC1 (*TOC1-*ox^24^) did not appear to cause a substantial change in time of greatest glyphosate sensitivity compared with the wild type (Fig. 1c; significant interaction between treatment and time; *P* = 0.04). The greatest decrease in hypocotyl length caused by glyphosate in the Col-0 wild type (26%) was smaller than the greatest decrease in *CCA1*-ox (46%) and more than the greatest decrease in *TOC1*-ox (15%) (Fig. 1a–c). These differing responses of *CCA1*-ox and *TOC1*-ox to glyphosate might reflect differences in their elongation response to light compared with the wild type^25^. Reduction of hypocotyl length was caused by glyphosate, not the herbicide adjuvants (Supplementary Fig. 3E). Together, this indicates that the circadian oscillator underlies a daily rhythm in glyphosate efficacy under light/dark cycles.

We reasoned that this daily cycle in the effectiveness of glyphosate might be due to a transient effect of glyphosate upon hypocotyl elongation. To test this, we used time-lapse imaging to monitor the rate of hypocotyl elongation following glyphosate treatment under light/dark cycles. After glyphosate treatment at dawn, the mean hypocotyl elongation rate was reduced significantly compared with the control during the first dark period after glyphosate treatment (Supplementary Fig. 4A, B). In contrast, the mean hypocotyl elongation rate was not decreased during this period when seedlings were treated with glyphosate at dusk (Supplementary Fig. 4C, D). Although the data are noisy, this suggests that glyphosate causes a transient reduction in hypocotyl elongation and the occurence of this transient reduction depends upon the time of glyphosate treatment.

We identified daily fluctuations in the effective dose of glyphosate. Increasing the dusk glyphosate concentration to 150 g/ha caused an equivalent reduction in hypocotyl length as 100 g/ha at dawn (Fig. 1d). Therefore, using the decrease in hypocotyl length as a measure of glyphosate effectiveness, 1.5 times more glyphosate was required at dusk to have the same effectiveness as at dawn. This suggests that plant growth is less sensitive to a dusk glyphosate application.

### Circadian regulation of a response to glyphosate

**Fig. 2:**
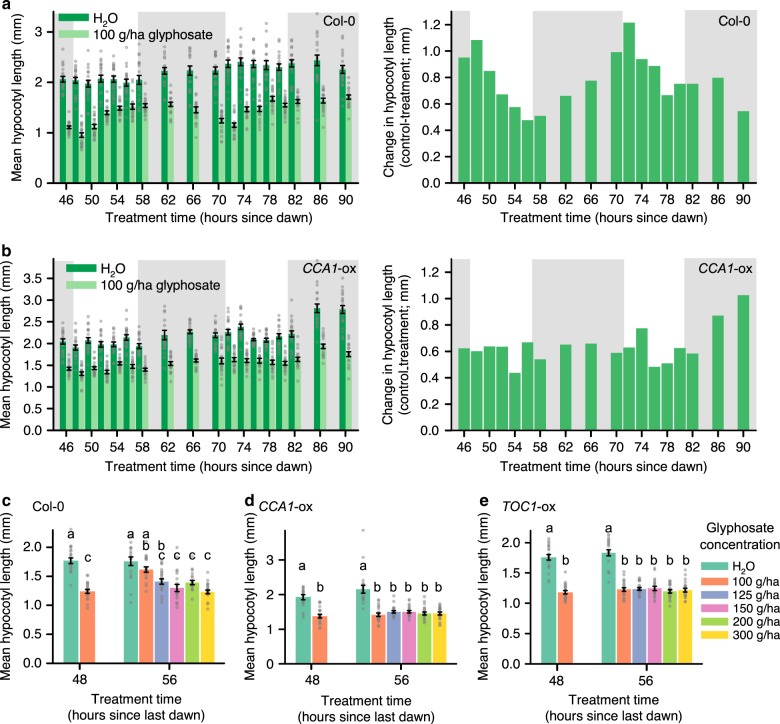
Circadian regulation of the sensitivity of plants to glyphosate. **a, b** Under constant light conditions, hypocotyl length of seedlings treated with 100 g/ha glyphosate at times specified, in **a** Col-0 and **b** *CCA1*-ox seedlings. Graphs show (left) hypocotyl length and (right) change in hypocotyl length caused by glyphosate. Grey shading indicates subjective night. **c–e** Glyphosate concentration required to produce equivalent attenuation of hypocotyl length at subjective dawn (48 h) and subjective dusk (56 h) in **c** Col-0, **d** *CCA1-*ox and **e** *TOC1-*ox. Significance determined by **a, b** two-way ANOVA **c**–**e** one-way ANOVA and post-hoc Tukey analysis. Different letters indicate significant differences between means. Data are mean ± s.e.m; *n* = 18–20. Source data are provided in the Source Data file

Next, we identified a circadian rhythm in the response of hypocotyl length to glyphosate. First, we verified that germinating seedlings receiving one 8 h light/16 h dark cycle had a free-running circadian rhythm, by measuring rhythms of *CCA1::LUCIFERASE* bioluminescence in seedlings receiving this treatment (Supplementary Fig. 5A, B; period 24.2 ± 0.1 h). The phase of *CCA1::LUCIFERASE* was consistent with previous reports under free running conditions^26–28^, with promoter activity increasing before subjective dawn and peaking around the middle of the subjective day (Supplementary Fig. 5A, B). Sets of seedlings were treated with 100 g/ha glyphosate at intervals across two 24 h cycles, beginning 46 h after the onset of constant conditions. The wild type had a circadian rhythm in the decrease in hypocotyl length caused by glyphosate (Fig. 2a, Supplementary Fig. 5C). Greatest glyphosate sensitivity occurred at subjective dawn, similar to the phase of maximum glyphosate sensitivity of hypocotyl length under light/dark cycles (Fig. 1a). In *CCA1-*ox, there was no rhythm in the decrease in hypocotyl length caused by glyphosate (Fig. 2b, Supplementary Fig. 5D). This identifies that the circadian oscillator underlies a circadian rhythm in the response of hypocotyl length to glyphosate.

Under constant light conditions, in the wild type 125 g/ha glyphosate was required at subjective dusk to attenuate hypocotyl length by the same magnitude as 100 g/ha glyphosate applied at subjective dawn (Fig. 2c). In contrast, in both *CCA1-*ox and *TOC1-*ox, glyphosate caused equivalent attenuation of hypocotyl elongation regardless of the glyphosate concentration or application time (Fig. 2d, e). Therefore, there is a circadian rhythm in the minimum effective dose of glyphosate that is controlled by the circadian oscillator.

### Rhythmic glyphosate sensitivity and auxin signalling

**Fig. 3:**
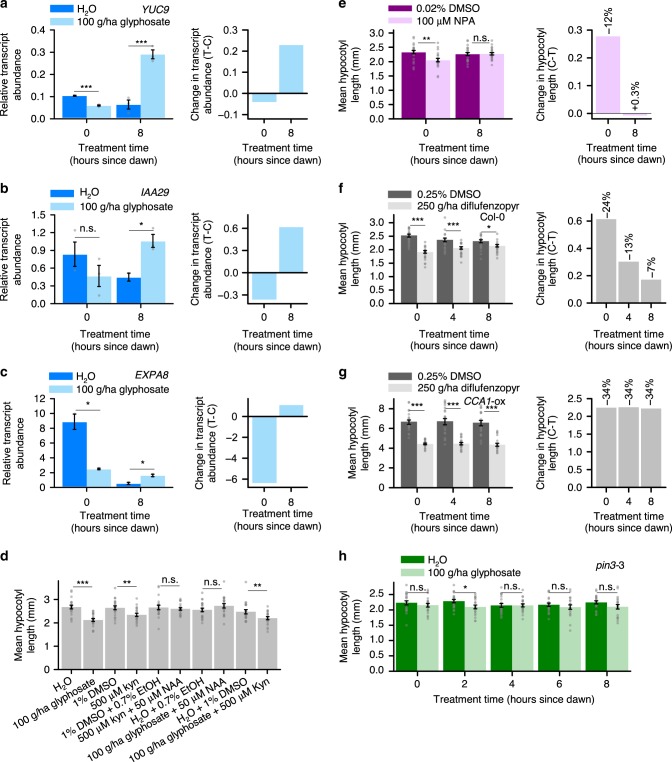
Processes associated with auxin signalling might underlie rhythmic sensitivity of hypocotyl length to glyphosate. **a**–**c** 100 g/ha glyphosate-induced alterations in auxin signalling transcript abundance depends on treatment time. Graphs show (left) relative transcript abundance and (right) change in transcript abundance caused by glyphosate. Reference transcript was *PP2AA3*. **d** Mean hypocotyl length of seedlings treated with either glyphosate, kynurenine (kyn), kyn + NAA, glyphosate + NAA, or glyphosate + kyn at dawn. **e** Mean hypocotyl lengths of seedlings treated with NPA or DMSO vehicle control at dawn or dusk. **f, g** Attenuation of hypocotyl length by 250 g/ha diflufenzopyr or DMSO vehicle control applied at 4 h intervals, in **f** Col-0 and **g** *CCA1*-ox seedlings under 8 h photoperiods. Chemical treatments were on day 3 and measurements on day 7. **g** Mean hypocotyl length of *pin3*-3 seedlings treated with 100 g/ha glyphosate at 2 h intervals throughout the photoperiod. Significance determined by **a**–**c**; **e**–**h** two-way ANOVA and *t*-test comparisons and **d** *t*-tests. n.s. = not statistically significant, *P ≤ 0.05, **P ≤ 0.01, ***P ≤ 0.001. Data are mean ± s.e.m: *n* = 2–3 (transcript analysis); *n* = 20 (hypocotyl measurements). Transcript abundance change expressed as treated (T) – control (**c**). Source data are provided in the Source Data file

We identified that auxin signalling-related transcripts have a rhythmic response to glyphosate. We examined the transcript abundance of *YUCCA9* (*YUC9*), which encodes a protein involved in an auxin biosynthesis pathway^29,30^, *INDOLE-3-ACETIC ACID INDUCIBLE29* (*IAA29*)^31^, which is induced rapidly by auxin, and *EXPANSIN A8* (*EXPA8*), which encodes an auxin-induced cell wall-modifying enzyme involved in turgor-driven cell expansion^32,33^. Glyphosate applied at dawn significantly decreased the transcript abundance of *YUC9* and *EXPA8*, but not *IAA29* (Fig. 3a–c). Furthermore, glyphosate applied at dusk significantly increased the abundance of *YUC9*, *IAA29* and *EXPA8* transcripts (Fig. 3a–c). The decrease in *YUC9* and *EXPA8* transcript abundance in response to glyphosate applied at dawn might suggest that auxin signalling becomes downregulated in response to glyphosate applied at this time, potentially explaining the greater sensitivity of hypocotyl elongation to glyphosate applied at dawn.

We found that the daily rhythm in the sensitivity of hypocotyl length to glyphosate might derive from inhibition of processes upstream of auxin signalling. Under light/dark cycles, the auxin biosynthesis inhibitor l-kynurenine^34^ caused the greatest attenuation of hypocotyl elongation when applied at dawn (Supplementary Fig. 6A, B), mimicking the timing of maximum sensitivity of elongation to glyphosate (Fig. 1a). Exogenous auxin (1-naphthaleneacetic acid; NAA) rescued the decrease in hypocotyl length caused by both kynurenine and glyphosate (Fig. 3d, Supplementary Fig. 6C), and glyphosate and kynurenine in combination did not cause additive reductions in hypocotyl length (Fig. 3d). One explantion for this result is that glyphosate and kynurenine might be acting upon the same pathway to alter hypocotyl length. As with glyphosate, the commonly used inhibitor of polar auxin transport 1-*N*-naphthylpthalamic acid (NPA)^35^ decreased hypocotyl length when applied at dawn but not when applied at dusk (Fig. 3e; two-way ANOVA identified statistically significant interaction of treatment with time; *P* = 0.02).

Because we identified differences between the length of hypocotyls following topical NPA application at dawn and dusk, we investigated the temporal response of hypocotyl length to diflufenzopyr. This herbicide active ingredient is thought to inhibit auxin transport^36^. We performed this experiment because we were interested to know whether there might be daily rhythms of sensitivity to herbicide active ingredients that inhibit aspects of auxin signalling. As with glyphosate, at the end of the experiment hypocotyls were shorter following 250 g/ha diflufenzopyr applied at dawn compared the hypocotyl length following diflufenzopyr applied at dusk (Fig. 3f; two-way ANOVA identifies a significant interaction between the treatment and time; *P* ≤ 0.001). Therefore, the magnitude of the change in hypocotyl length in response to diflufenzopyr depends on treatment time even though diflufenzopyr causes a significant reduction in hypocotyl length at all times. In comparison, in *CCA1*-ox diflufenzopyr application reduced the hypocotyl length at all times tested, but the magnitude of decrease in hypocotyl length did not depend on the time of diflufenzopyr application (Fig. 3g; *P* *=* 0.99, two-way ANOVA). Taken together, these data suggest that inhibition by glyphosate of processes related to auxin signalling might underlie the rhythmic sensitivity of hypocotyl length to glyphosate. This inhibition of auxin signalling by glyphosate might be indirect because auxin biosynthesis shares initial steps with the biosynthesis of aromatic amino acids, which is suppressed by glyphosate.

We identified that auxin transport has potential involvement in the rhythmic sensitivity of elongating hypocotyls to glyphosate. PIN-FORMED3 (PIN3) participates in polar auxin transport within hypocotyls^37–39^, and *PIN3* transcripts are rhythmic and glyphosate-responsive (Supplementary Data 1). In *pin3*-3, the hypocotyl length at the end of the experiment was not altered significantly in response to glyphosate applied at most times tested during the light period of light/dark cycles. Furthermore, the daily rhythms of glyphosate sensitivity of hypocotyl length that occur in the wild type were absent from *pin3*-3 (Fig. 3h; *P* = 0.69, two-way ANOVA). Therefore, PIN3-mediated auxin transport might be required for rhythmic sensitivity of elongating hypocotyls to glyphosate under light-dark cycles. This was surprising, because PIN3 is reported to have functional redundancy with PIN1 and PIN7 in elongating hypocotyls^40,41^. Other auxin biosynthesis mutants, such as *yuc1 yuc2 yuc4 yuc6*, *wei8 tar2* and *rooty* are impractical for this type of experiment because it is problematic to work with segregating populations in this type of assay. We also found that long hypocotyls of glyphosate-treated *phyB* arise from the long hypocotyl phenotype of *phyB*^42^ rather than proposed glyphosate resistance^8^ (Supplementary Fig. 7A–D).

### Circadian and diel responses of cell death to glyphosate

**Fig. 4:**
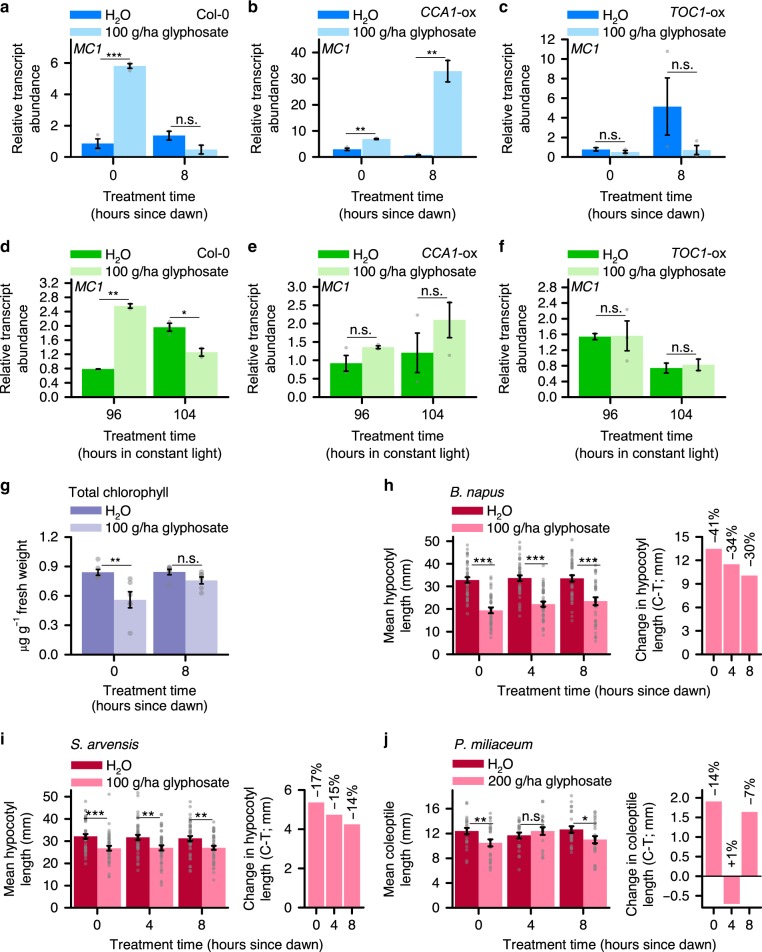
Impact upon plants of daily and circadian rhythms of glyphosate efficacy. **a**–**f** 100 g/ha glyphosate alters programmed cell death transcript *MC1* in a time of day-dependent manner under both light-dark cycles (**a–c**) and constant light (**d**–**f**). Reference transcript was *PP2AA3*. **g** 100 g/ha glyphosate alters total chlorophyll content depending on the time of day of application. **h**–**j** Diel changes in sensitivity of hypocotyl or coleoptile length to glyphosate in agriculturally relevant species. **h, i** Mean hypocotyl length of *B. napus* and *S. arvensis* and treated with 100 g/ha glyphosate and **j** mean coleoptile length of *P. miliaceum* treated with 200 g/ha glyphosate. Graphs show (left) hypocotyl/coleoptile length and (right) hypocotyl/coleoptile length change caused by glyphosate. A greater glyphosate concentration was required to alter coleoptile length in *P. miliaceum* (200 g/ha) compared with hypocoyl length in the dicots (100 g/ha), possibly because monocots and dicots have differing auxin responses^80^. Significance determined by two-way ANOVA and *t*-test comparisons between control and treatment at each timepoint. n.s. = not statistically significant, *P ≤ 0.05, **P ≤ 0.01, ***P ≤ 0.001. Data are mean ± s.e.m.; *n* = 2–3 (transcript analysis), *n* = 20–45 (data from multiple experiments, hypocotyl measurements). Source data are provided in the Source Data file

We found that markers of cell death have rhythmic responses to glyphosate. Under light/dark cycles, glyphosate applied to the wild type at dawn but not dusk significantly increased the abundance of transcripts encoding the positive regulator of cell death *METACASPASE1* (*MC1*)^43^ (Fig. 4a; significant interaction between glyphosate treatment and application time, *P* < 0.001). In contrast, *MC1* transcripts were increased by glyphosate at both dawn and dusk in *CCA1*-ox (Fig. 4b; significant interaction between glyphosate treatment and application time, *P* < 0.001), and glyphosate did not increase *MC1* transcripts in *TOC1*-ox when applied at either dawn or dusk (Fig. 4c; *P* = 0.3).

Under constant light conditions, glyphosate significantly increased the abundance of *MC1* transcripts when applied at subjective dawn, whereas *MC1* transcript abundance was reduced by glyphosate application at subjective dusk (Fig. 4d; significant interaction between glyphosate treatment and application time *P* < 0.001). In contrast, *MC1* transcripts did not increase in *CCA1*-ox or *TOC1*-ox when glyphosate was applied at either subjective dawn or subjective dusk (Fig. 4e, f). We also investigated the response to glyphosate of the negative regulator of cell death (*DEFENDER AGAINST APOPTOTIC DEATH1* (*DAD1*)^44^), but found that *DAD1* did not respond to glyphosate under our experimental conditions in the genotypes that we tested (Supplementary Fig. 7E–J). Overall, the response of *MC1* transcripts to glyphosate is consistent with the notion that glyphosate applied to wild type plants at dawn or subjective dawn might cause greater cell death than when applied at dusk or subjective dusk (Fig. 4a–f).

Another marker for cell death is a reduction in the concentration of chlorophyll^45^, with chlorophyll degradation also being induced by glyphosate^8,46,47^. Under light-dark cycles, glyphosate application to wild type plants at dawn but not dusk significantly decreased the chlorophyll concentration (Fig. 4g). In addition to ROS and lipid peroxidation^47^, decreased abundance of transcripts encoding *GENOMES UNCOUPLED4* (*GUN4*) (Supplementary Data 1)^15^ might represent a mechanism by which glyphosate reduces chlorophyll concentration, because *gun4* mutants have reduced chlorophyll accumulation^48^. Taken together, two indicators of cell death (*MC1* transcripts and chlorophyll concentration) report that glyphosate application at dawn causes cell death more rapidly than when applied at dusk, with circadian regulation potentially underlying this response.

### Species-specificity of rhythmic responses to glyphosate

Rhythmic developmental responses to glyphosate also occur in certain agriculturally relevant species. In dicotyledonous *Brassica napus* and *Sinapis arvensis*, glyphosate caused a significant decrease in hypocotyl length. Although a dawn glyphosate application caused a greater reduction in hypocotyl length compared with a dusk glyphosate application, the interaction between glyphosate and treatment time was not statistically significant (Fig. 4h, i; *P* = 0.45 and *P* = 0.87 from two-way ANOVA for *B. napus* and *S. arvensis*, respectively). In contrast, in monocotyledonous *Panicum miliaceum*, glyphosate applied at dawn and dusk caused a significant decrease in coleoptile length, but not when applied in the middle of the photoperiod (Fig. 4j; *P* = 0.05 from two-way ANOVA) (monocot coleoptile elongation is also auxin-regulated^49^). This identifies that under laboratory conditions, there are species-dependent differences in the time of day variation in the response of seedling growth to glyphosate.

## Discussion

We identified that the circadian clock controls the sensitivity of plant development and cell death to glyphosate. With the exception of varieties that have evolved glyphosate tolerance, glyphosate is generally not degraded by plants^50,51^ but appears to have transient effects that underlie its rhythmic efficacy (e.g. Supplementary Fig. 4). In nature, microbial metabolism of glyphosate^52,53^ might alter or enhance these transient effects compared with the data that we obtained under sterile laboratory conditions. In the field, fluctuations in glyphosate translocation, leaf angle and environmental conditions might contribute to daily rhythms of glyphosate effectiveness^5–7^. Furthermore, environmental conditions can also determine the time when growers decide to spray. Transport processes and leaf position can be circadian-regulated in plants^54,55^, so it is possible that circadian regulation contributes to plant glyphosate responses through multiple mechanisms. In future, it will be informative to identify the exact mechanism by which glyphosate might attentuate hypocotyl elongation and/or auxin signalling, and the extent to which this could scale to an agricultural context.

There are circadian rhythms of stomatal opening that are regulated by the circadian oscillator^10,56^, but we think it unlikely that such rhythms of stomatal opening contribute to daily cycles of glyphosate sensitivity in our experiments. Firstly, in Arabidopsis under laboratory conditions of light/dark cycles and constant light the stomata reach peak conductance around the middle of the day^10,57^, whereas peak glyphosate sensitivity occurred around dawn (Fig. 1a; Fig. 2a). Therefore, the phase of maximum stomatal conductance and maximum glyphosate sensitivity appear to be different. Second, because our experiments used agar-grown seedlings enclosed within petri dishes in growth chambers to provide a consistent and reproducible experimental environment, the daily fluctuations in humidity, growth medium moisture and temperature that cause midday stomatal closure were absent. Finally, herbicide formulations tend to enter leaves through the stomatal flooding only when combined with an organosilicone superwetter such as Silwet L77^58,59^, which is commercially uncommon due to cost. The adjuvant within the formulation that we used was a standard mix of alkylamine ethoxylate and a co-surfactant, which will cause cuticular rather than stomatal uptake. The very narrow capillary formed by the stomatal pore combined with surface tension generally prevents stomatal entry of water, otherwise leaves would flood when it rains.

Overall, our data indicate that there is circadian regulation of the sensitivity of plants to chemicals that affect biological processes, in a manner comparable to rhythmic responses to drugs in mammals^13^. This could extend the concept of chronotherapy to agriculture. The pervasive influence of circadian regulation upon plant metabolism suggests that the principle we identify might scale to other agrochemicals. The circadian regulation of plant responses to agrochemicals provides a basis to refine agrochemical development and use, through this novel concept of agricultural chronotherapy, to optimise crop protection for food security.

## Methods

### Identification of rhythmic glyphosate-responsive transcripts

Lists of transcripts that are either glyphosate-induced or repressed^15^ were compared with those that oscillate with a circadian or diel rhythm^11,16–19^ to identify transcripts that are both glyphosate- and circadian/diel-regulated. Statistical significance was determined using hypergeometric tests. Each of these transcripts was also assigned to bins of 4 h according to its circadian phase, using phase data from a previous report^18^. Gene descriptions were extracted using The Arabidopsis Information Resource (TAIR) (accessed 11/05/18). GO-term analysis of the set of glyphosate-responsive and rhythmic transcripts was performed using ThaleMine on the Araport platform using version 11 of the Arabidopsis genome (Araport11). The subset of overlapping glyphosate and circadian/diel regulated transcripts was compared with an auxin-related GO term list. For this comparison, 549 unique transcripts were identified from 33 different auxin-related GO terms in the Arabidopsis genome (Supplementary Data 1), using AmiGO2 (accessed 11/05/18).

### Glyphosate formulation

A glyphosate formulation (Touchdown Total, Syngenta) containing glyphosate and a proprietary adjuvant was used for this work. In agriculture, herbicide concentration is expressed as the mass of active ingredient per hectare (g/ha) based on a typical spray volume application rate of 200 l/ha, so we used this convention here. Glyphosate formulations that are used at the typical field rate of 840 g/ha contain 24.8 mM of glyphosate. Since our experiments involved the topical application of glyphosate to agar-cultivated seedlings several days after germination, we measured the dose response of hypocotyl length to glyphosate to select a suitable experimental concentration. We selected a concentration of glyphosate for the majority of our experiments (100 g/ha, equivalent to 2.95 mM; Supplementary Fig. 8A) that caused partial attenuation of hypocotyl elongation, because we reasoned that this would allow us to detect potential daily variations the response of hypocotyl length to glyphosate (Supplementary Fig. 8A). The glyphosate concentration that we used (100 g/ha) is lower than a reported ED50 for glyphosate in Arabidopsis of 350 g/ha^60^.

### Plant material and growth conditions

Unless stated otherwise, seeds were surface sterilised with 70% (v/v) ethanol for 1 min, 20% (v/v) domestic bleach for 12 min, followed by two washes with sterile distilled H_2_O. Seeds were subsequently re-suspended in 0.1% (w/v) agar for pipetting. Growth media comprised half-strength (2.15 g l^−1^) Murashige and Skoog nutrient mix (basal salts without vitamins, Duchefa Biochimie, Haarlem, Netherlands; pH 6.8) and 0.8% (w/v) agar, using sterile plastic rings embedded in media to allow equal dosing of seedlings with chemicals. Approximately 12 seeds were sown per ring. Seeds were stratified in darkness for 3 days at 4 °C before transfer to growth chambers (8 h light/16 h dark, 12 h light/12 h dark, or 16 h light/8 h dark; 19 °C, ~100 µmol m^−2^ s^−1^ photon flux density; MLR-352 chambers, Panasonic, Osaka, Japan). Genotypes used were Col-0, L. *er*, *CCA1*-ox^23^, *TOC1*-ox^24^, *pin3*-3^61^, *phyB*^62,63^ and *CCA1::LUCIFERASE*^64^.

To examine the effect of glyphosate on mature Arabidopsis plants (Supplementary Fig. 1), seedlings grown as above were transplanted after 7 days onto compost (Levington Advance F2 Seed & Modular, ICL) and transferred into a different growth chamber (12 h light/12 h dark, 19 °C, ~100 µmol m^−2^ s^−1^ photon flux density; 70% relative humidity; Snijders Labs Micro Clima-Series). Plants were treated with the recommended field rate of glyphosate (840 g/ha) at the six true leaf stage and photographed 14 days later (Nikon D80 DSLR).

### Hypocotyl length measurement

Seeds were prepared as above, with two plastic rings on each petri dish and two petri dishes per chemical treatment per time point. Plants were treated on day 3 or day 5 after germination (for each plastic ring of 12 seedlings, this comprised 20 µL 100 g/ha glyphosate, water control, or other treatment) at specified time points, and returned to the growth cabinet. 4 days after treatment, 18–25 plants were measured per treatment, per time point by positioning plants on 1% (w/v) agar and taking photographs, followed by manual analysis of hypocotyl length using the image analysis programme ImageJ^65^. Hypocotyls were measured from the shoot apical meristem to the shoot–root junction. We treated seedlings with glyphosate on day 3 and measured hypocotyl length on day 7 after germination because the intervening period is one of rapid hypocotyl elongation^22^, therefore providing the opportunity to detect variation in the impact of glyphosate upon hypocotyl elongation. Hypocotyls ranged 2–4 mm under our short day conditions (8 h photoperiod, 100 μmol m^−2^ s^−1^ photon flux density; 19 °C), which is positioned within the range of hypocotyl lengths (2–7 mm) reported for Arabidopsis seedlings under short day conditions^66–69^. Hypocotyl length was not altered by either topical addition of liquid to the seedlings, nor the presence of plastic rings embedded within the media that were used to direct the chemical application (Supplementary Fig. 8B). Hypocotyls were measured as one batch, rather than staggered according to the time that they were treated with glyphosate. Hypocotyl length was measured at approximately dawn on day 7 after germination. We verified that irrespective of whether the hypocotyl length was measured in one batch or staggered according to time of treatment, hypocotyl elongation was attenuated more in response to glyphosate treatment at dawn than at dusk (Supplementary Fig. 8C, D). Exact numbers of replicate plants for each treatment and experiment are provided in Supplementary Data 2. For clarity, many figures show actual hypocotyl length, the change in hypocotyl length resulting from glyphosate treatment (calculated as the difference between the mean control hypocotyl length and mean treated hypocotyl length), and the proportional change in hypocotyl length.

For experiments with l-kynurenine (kyn; Sigma-Aldrich), kyn was applied using 1% (v/v) dimethyl sulfoxide (DMSO) as a vehicle control (for the highest volume DMSO). For experiments with 1-naphthaleneacetic acid (NAA; Sigma-Aldrich), seedlings were treated with 500 µM kyn supplemented with NAA. This concentration was consistent with the range of concentrations used by previous studies involving hypocotyl length measurements^70,71^. The vehicle control was 0.7% (v/v) EtOH + 1% (v/v) DMSO. For experiments with diflufenzopyr (Syngenta), 250 g/ha was chosen because it caused similar attenuation in hypocotyl length as 100 g/ha glyphosate, using 0.25% (v/v) DMSO as a vehicle control (Supplementary Fig. 8E). The concentration range of NAA used (1–100 μM) was consistent with other studies^72–74^. For experiments with 1-*N*-naphthylphthalamic acid (NPA; Sigma), 100 μM NPA was chosen because this concentration gave a similar level of attenuation in hypocotyl length as glyphosate and this concentration has been used reliably by other studies^35,75,76^. 0.02% (v/v) DMSO was used as a vehicle control for NPA. To investigate whether the adjuvant within the glyphosate formulation affected hypocotyl elongation (Supplementary Fig. 3B), a glyphosate adjuvant control formulation was produced (Syngenta). This control formulation was used at the equivalent mass of adjuvant present within the 100 g/ha glyphosate treatment.

### Hypocotyl elongation rate measurement

To measure the rate of hypocotyl elongation, seeds were sown individually onto petri dishes which, after stratification, were positioned vertically within the growth chamber. Chemical treatments occurred at either dawn or dusk on day 3 after germination, and imaging commenced after the chemical treatment. 1.6 µL of either water or 100 g/ha glyphosate was applied to each seedling, which was an equivalent volume to other treatments. Time lapse images were captured with a Nikon D80 DSLR with its infra-red (IR) blocking filter removed, and replaced with an IR pass filter (>850 nm) (Zomei, Jiangsu, China). Plates were backlit with a custom-built IR LED array (880 nm) to allow images to be captured in darkness. Images were captured every 30 min following chemical treatment, for 96 h. Ten seedlings were measured per treatment. Hypocotyl lengths were measured manually from the time-series using ImageJ. The physical differences between the apparatus used to measure elongation rates and perform endpoint hypocotyl length measurements means that data such as the magnitude of changes in hypocotyl length over time is not identically comparable between the two assay types.

### Investigation of circadian rhythms in emerging seedlings

Stratified Col-0 *CCA1::LUCIFERASE* seeds were placed into 8 h light/16 h dark conditions for 1 day, and then transferred to continuous light. 24 h before imaging, seeds were dosed with 100 µL 5 mM luciferin (potassium salt of D-luciferin; Melford Laboratories Ltd). Luciferase bioluminescence was imaged for 6 days using a Lumintek EM-CCD imaging system (Photek) controlled by Image32 software (Photek). This involved 45-s integrations of the bioluminescence signal at hourly intervals, with the EM gain on the camera set to 2700. This was followed by analysis of rhythmic features within the data using the fast Fourier transform-nonlinear least-squares (FFT-NLLS) algorithm within BRASS software (millar.bio.ed.ac.uk; University of Edinburgh).

### Effect of glyphosate on transcript abundance

To measure transcript abundance, seedlings were cultivated as above. Aerial tissue was sampled 3 h (*YUC9*, *IAA29*, *EXPA8*) or 6 h (*MC1*, *DAD1*) after chemical treatment. For each RNA sample, the aerial tissue of ~20 seedlings was harvested from across two petri dishes of seedlings (i.e. about ten seedlings from each petri dish). RNA was isolated using the Nucleospin II RNA extraction kit (Machery-Nagel), with subsequent cDNA synthesis conducted with the High-Capacity cDNA Synthesis kit (Thermo-Fisher), both according to manufacturer’s instructions. qRT-PCR analysis was performed using HOT FIREPol EvaGreen qPCR reagents (Solis BioDyne) and Agilent Mx3005P qPCR instrument. Amplification efficiency was determined from individual PCR reactions using a linear regression on the straight-line portion of log(fluorescence) during the PCR amplification, using LinRegPCR (version 2018.0)^77^. Ct was determined using a threshold Rn of 0.2. Transcript abundance was determined relative to *PP2AA3* as a reference transcript^66^ using a calculation (Equation 1) that incorporates the efficiency of each amplification^28,78^. We verified that *PP2AA3* reference transcript amplification was unaltered by glyphosate treatment (control Ct = 27.17 ± 0.10; glyphosate Ct = 27.05 ± 0.09; *n* = 6; *P* = 0.37 from two-sample *t* test). Primer sequences are provided in Supplementary Table 1.

1 $${\rm{RTA}}\left( {\rm{GOI}} \right) = \left( {{\rm{RE}}_{\rm{RG}}} \right)^{C_t\left( {\rm{RG}} \right)} \times \left( {{\rm{RE}}_{\rm{GOI}}} \right)^{ - C_t\left( {\rm{GOI}} \right)}.$$

Determination of relative transcript abundance. Calculation determines relative transcript abundance of gene of interest (GOI), where RE_RG_ and RE_GOI_ are the mean amplification efficiencies of the reference gene and gene of interest for each sample, and *C*_*t*_*(*RG*)* and *C*_*t*_*(*GOI*)* are the mean *C*_*t*_ values for the reference gene and gene of interest for each sample, respectively.

### Chlorophyll content analysis

Total chlorophyll was extracted from elongating hypocotyls four days after glyphosate treatment, at either dawn or dusk, with 80% (v/v) buffered aqueous acetone^79^. Total chlorophyll (chlorophyll *a* and *b*) was calculated as by Porra et al. (Eq. 2)^79^.

2 $${\rm{Chl}}_{\rm{total}}\left( {\rm{mg}}\,{\rm{g}}^{ - 1} \right) = \frac{{V\left( {17.76A}^{646} + {7.34A}^{663} \right)}}{{1000W}}$$

Determination of total chlorophyll content. *V* is the volume of 80% (v/v) acetone, *A* is the absorbency at 646 nm and 663 nm, and *W* is the tissue weight in grams.

### Hypocotyl and coleoptile elongation in non-model species

Non-sterile seeds of rapeseed (*Brassica napus*), wild mustard (*Sinapis arvensis*) and proso millet (*Panicum miliaceum*) were placed onto water-saturated filter paper in petri dishes for 3 days (*S. arvensis, P. miliaceum*) or 5 days (*B. napus*) at room temperature under constant light. Seeds were then transplanted onto 3:1 compost and sand mixture and placed into growth chambers with conditions of 8 h light/16 h dark, 19 °C, ~100 µmol m^−2^ s^−1^ photon flux density; 70% relative humidity (Snijders Labs Micro Clima-Series chamber). After 3 days plants were sprayed with glyphosate (100 g/ha, or 200 g/ha for *P. miliaceum*) using a custom-built laboratory-sized track sprayer, at dawn, midday, or dusk. 4 days after treatments, plants were imaged and hypocotyls or coleoptiles were measured as in previous hypocotyl assays. Data shown are the mean of multiple experimental repeats.

### Data analysis

Statistical analysis was performed with Sigmaplot 13.0, except hypergeometric tests were performed in Excel to investigate the intersection of transcriptomes. Fast Fourier transform (non-linear least squares method) (FFT-NLLS) analysis of bioluminescence imaging data was performed using BRASS (millar.bio.ed.ac.uk; University of Edinburgh). Output from all statistical analyses, including sample sizes and *P* values, are provided in Supplementary Data 2.

### Reporting summary

Further information on research design is available in the Nature Research Reporting Summary linked to this article.

## Supplementary information

Supplementary Information

Description of Additional Supplementary Files

Supplementary Data 1

Supplementary Data 2

Reporting Summary

Source Data

## Data Availability

All relevant data are included in the figures, supplementary information files, and Source Data file. The source data underlying Figs. 1–4 and Supplementary Figs. 3–8 are provided as a Source Data file. Bioinformatics and statistical analyses are in Supplementary Data 1 and Supplementary Data 2, respectively.
