# Supplementary material for: Plant circadian rhythms regulate the effectiveness of a glyphosate-based herbicide
Source: Nat Commun. 2019 Aug 16;10:3704. doi: 10.1038/s41467-019-11709-5 (PMC6697731; doi:10.1038/s41467-019-11709-5)
Supplement: Supplementary file 1 — Supplementary Information [file 41467_2019_11709_MOESM1_ESM.pdf]

Belbin et al. Supplementary Materials

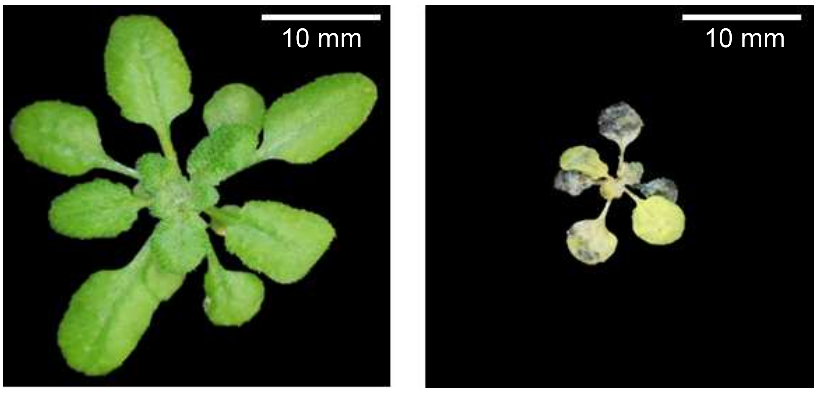

Control 840 g/ha glyphosate

Supplementary Figure 1. Representative effect of field rate of glyphosate applied to Arabidopsis rosettes. Images taken 14 days after either treatment with 840 g/ha glyphosate or water control. 10 mm scale bars indicated on figures.

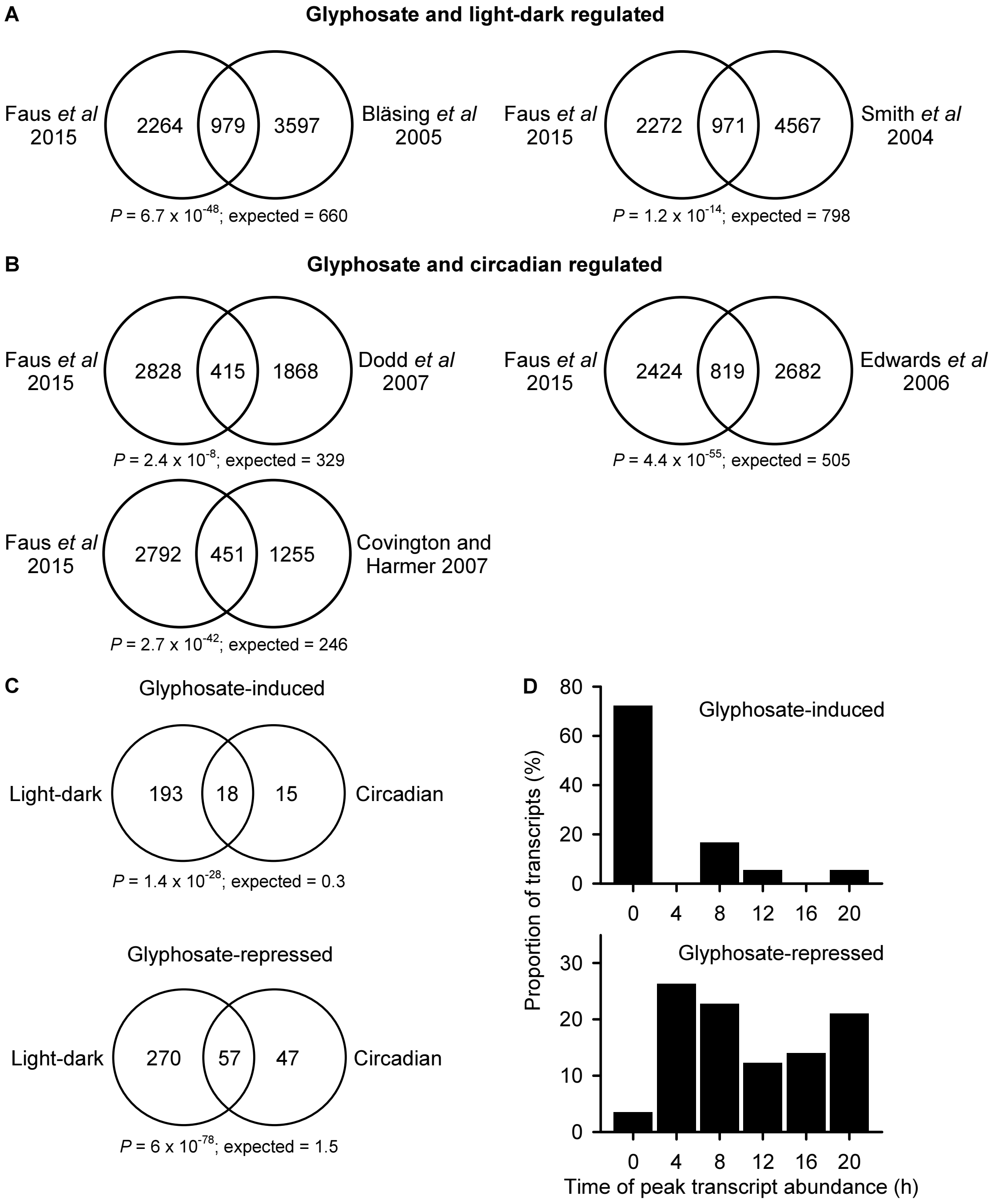

Supplementary Figure 2. Identification of rhythmic glyphosate-responsive transcripts within multiple circadian or diel transcriptomes. There are significant overlaps between glyphosate regulated and light-dark-regulated (A) or circadian-regulated (B) transcriptomes (*P* =6.7 x 10^{-48}, 1.2 x 10^{-14}, 2.4 x 10^{-08}, 4.4 x 10^{-55}, 2.7 x 10^{-42} respectively, determined by hypergeometric test). (C) A significant number of light-dark- and circadian-regulated transcripts are glyphosate-induced (*P* = 1.4 x 10^{-28}) and glyphosate-repressed (*P* = 6 x

10^{-78}). The statistical significance of each intersection being a chance overlap was determined by hypergeometric tests, and the number of transcripts that would be expected within a chance overlap of the transcript sets is provided below each Venn diagram. (D) Temporal clustering of rhythmic glyphosate-responsive transcripts, binned by phase of expression. Analysis combines transcripts having circadian and diel rhythms. Lists of genes are within Supplemental Dataset S1.

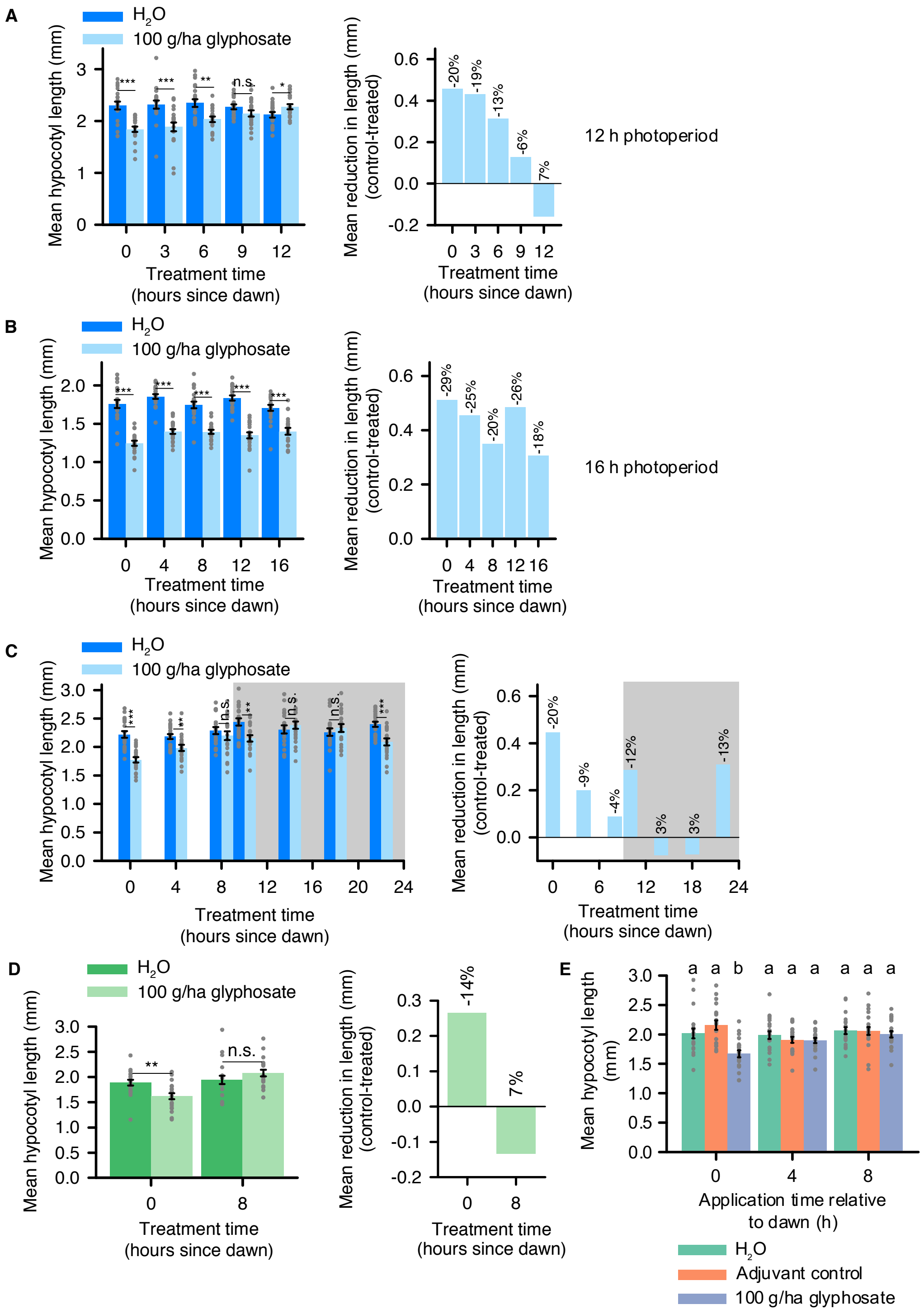

Supplementary Figure 3. Daily rhythms of glyphosate effectiveness under several photoperiodic conditions. (A-C) Under light/dark cycles, response of hypocotyl length to 100 g/ha glyphosate applied at times specified under (A) 12 h photoperiods; (B) 16 h photoperiods; (C) during the dark period under 8 h photoperiods; (D) following glyphosate

application on Day 5 after germination. Graphs show (left) hypocotyl length and (right) change in hypocotyl length caused by glyphosate, and glyphosate was applied on Day 3 (A-C) or Day 5 (D) after germination. Significance determined by two-way ANOVA and *t*-tests where * indicates $P \leq 0.05$; ** indicates $P \leq 0.01$ and *** indicates P < 0.001. (E) Glyphosate adjuvant is without effect upon hypocotyl elongation. Mean hypocotyl length of Col-0 seedlings treated with either 100 g/ha glyphosate, the equivalent mass of glyphosate adjuvant blank formulation, or water control, at three times through the 8 h photoperiod. In (E), different letters indicate statistically significant differences between means. Significance determined by one-way ANOVA followed by post-hoc Tukey analysis, performed separately for each time point. Data throughout are mean ± s.e.m; n = 20. Source data are provided in the Source Data file.

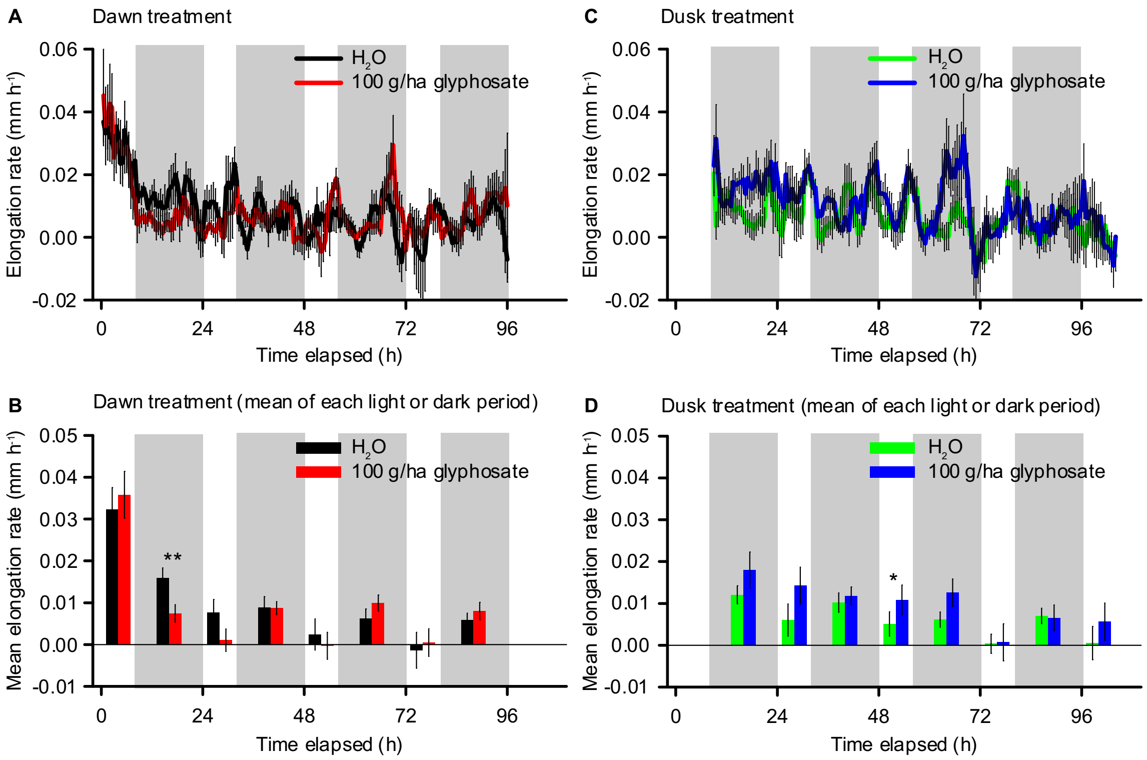

Supplementary Figure 4. Effect of glyphosate application at dawn or dusk upon the rate of hypocotyl elongation. (A, C) Mean hypocotyl elongation rate, measured using timelapse imaging of seedlings under 8 h photoperiods, following seedling treatment with glyphosate at (A) dawn and (C) dusk. Elongation rate was calculated as a 3 h rolling average. (B, D) Mean hypocotyl elongation rate during each light and dark period of each cycle, for seedlings treated with glyphosate at (B) dawn and (D) dusk. Values are mean ± s.e.m; n = 10. (B, D) Analysed by Mann-Whitney rank sum test where * indicates $P \leq 0.05$ and ** indicates $P \leq 0.01$. Source data are provided in the Source Data file.

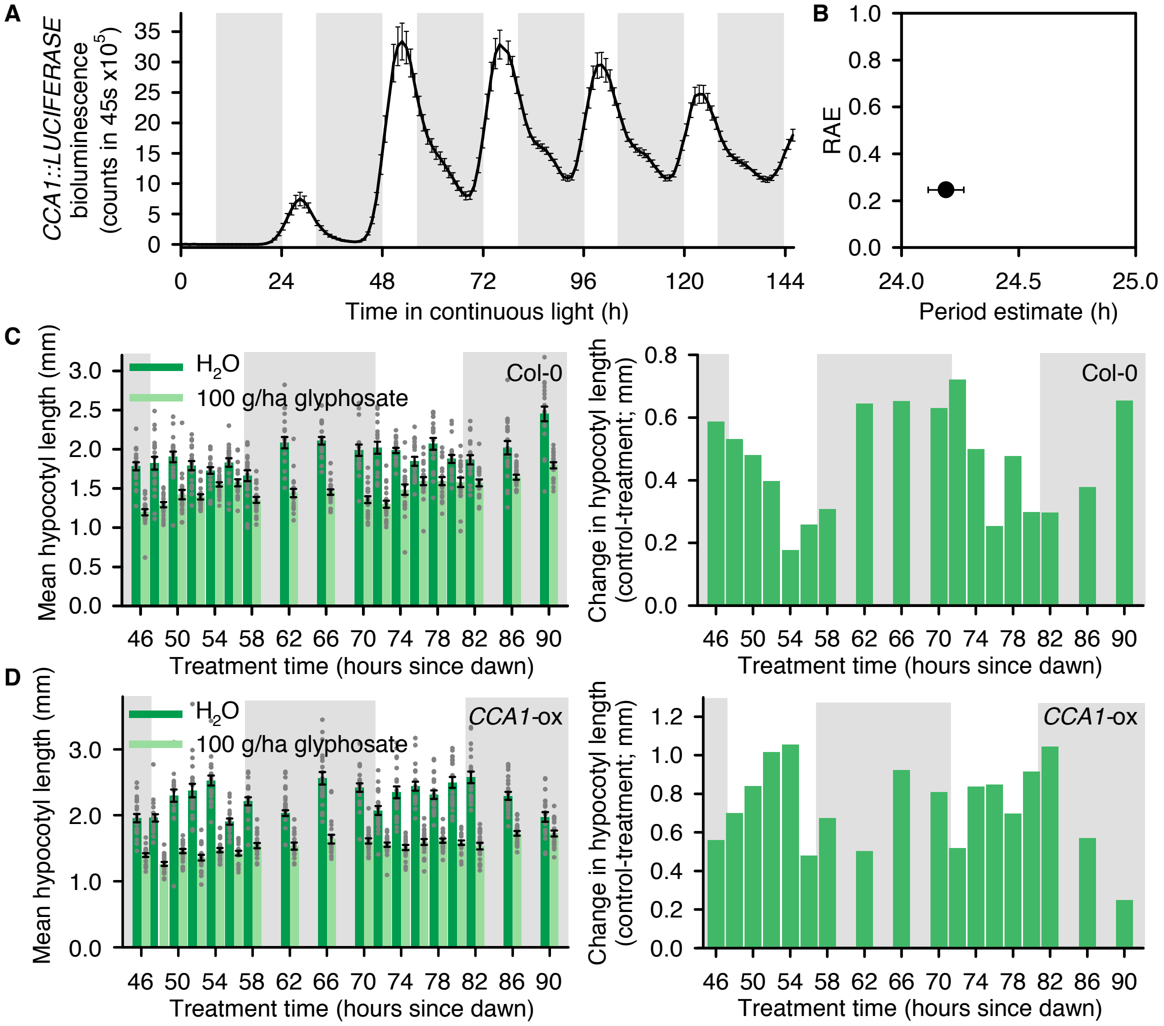

Supplementary Figure 5. Circadian oscillations in the sensitivity of hypocotyl length to glyphosate. (A) Circadian oscillations of *CCA1::LUC* bioluminescence in germinating seedlings and (B) comparison of the estimated period of the oscillation with the relative amplitude error (RAE). RAE is derived from analysis by fast Fourier transform-nonlinear least-squares method. (C, D) Direct repeat of experiment in Fig. 2E and F. Data are mean ± s.e.m; (A and B) *n* = 8 clusters of seedlings; (C, D) *n* = 20. Source data are provided in the Source Data file.

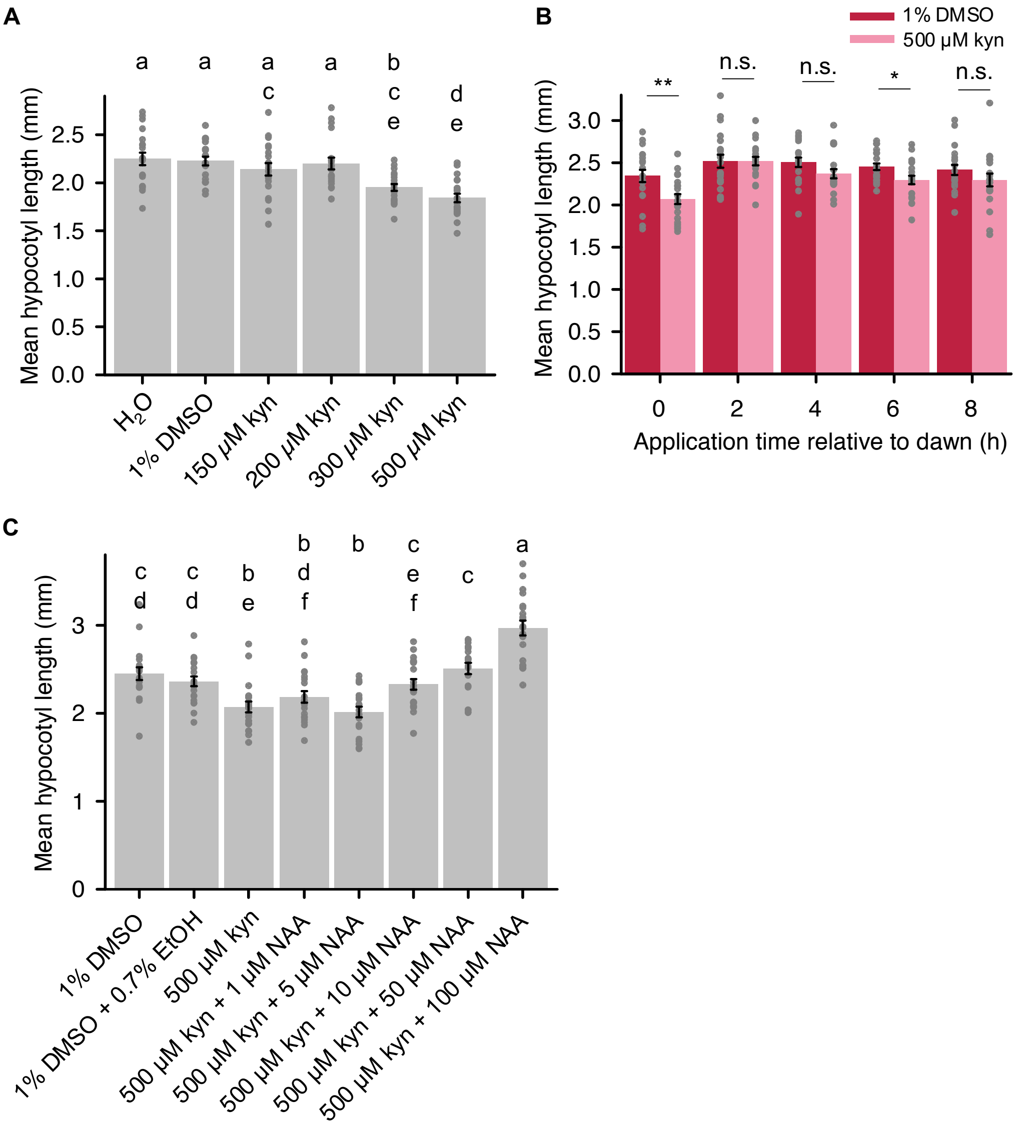

Supplementary Figure 6. Effect of auxin signalling agonists and antagonists upon the time of day sensitivity of elongating hypocotyls to glyphosate. (A) Mean hypocotyl length after treatment by a range of concentrations of L-kynurenine (kyn) at dawn. (B) Mean hypocotyl length after 500 μM L-kynurenine treatment at 2 h intervals throughout the 8 h day under L/D cycles. (C) Mean hypocotyl length after treatment with 500 μM L-kynurenine and various concentrations of NAA. Significance determined by (A, C) one-way ANOVA followed by post-hoc Tukey analysis and (B) two-way ANOVA with *t*-tests. n.s. = not statistically significant, * $P \leq 0.05$, ** $P \leq 0.01$, *** $P \leq 0.001$. Data are mean ± s.e.m; $n = 20$. Source data are provided in the Source Data file.

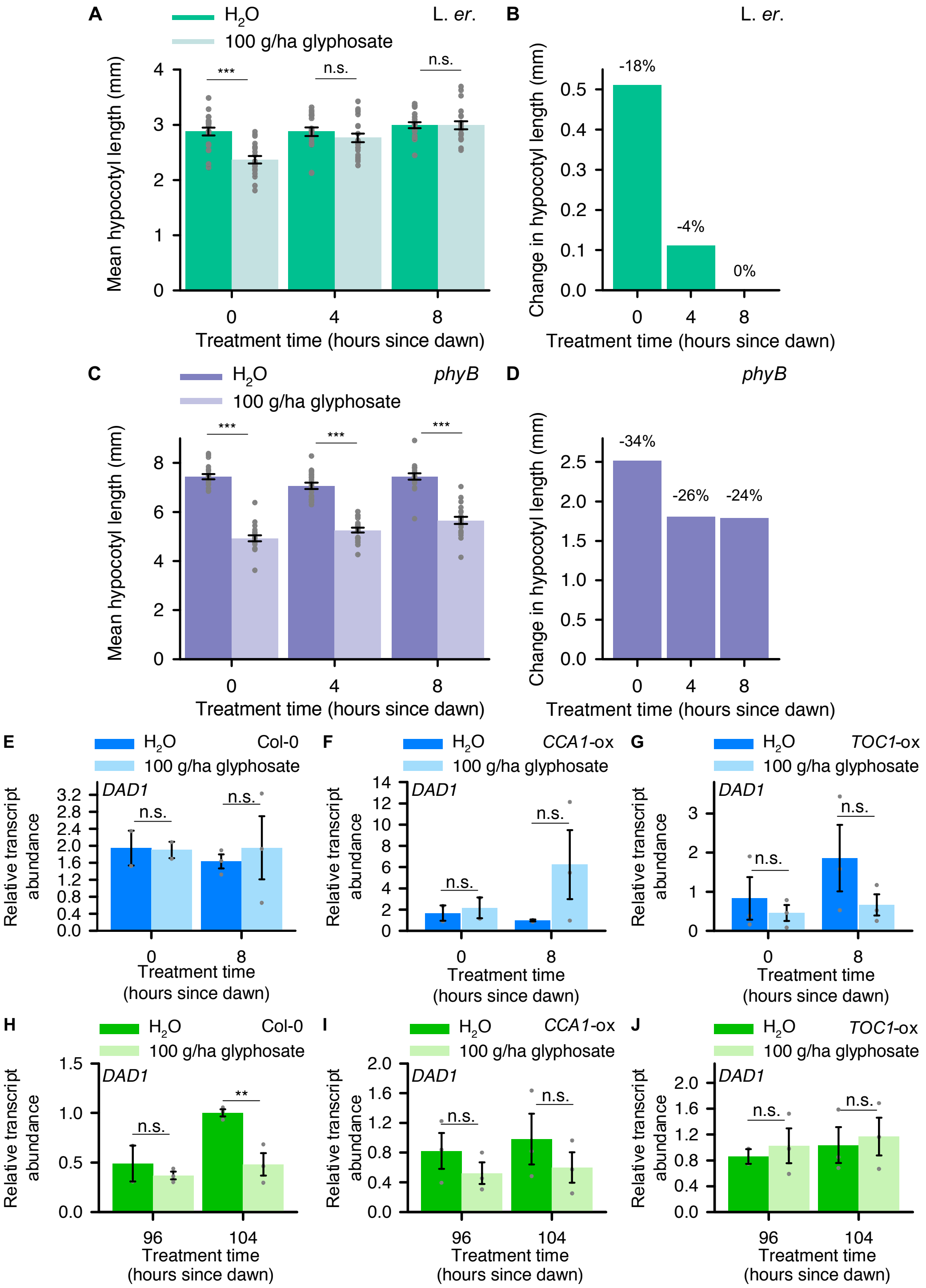

Supplementary Figure 7. Response of *phyB* mutant hypocotyls and *DAD1* transcripts to glyphosate. (A-D) *phyB* mutation does not confer glyphosate resistance to elongating hypocotyls. Attenuation of hypocotyl elongation in (A, B) L. *er* and (C, D) *phyB* seedlings treated with 100 g/ha glyphosate at dawn, midday and dusk under light/dark cycles. Data are

mean ± s.e.m; *n* = 20. (E-J) Response of negative regulator of programmed cell death *DAD1* to 100 g/ha glyphosate applied at (E-G) dawn or dusk under light/dark cycles and (H-J) subjective dawn or dusk under constant light conditions. *PP2AA3* is reference transcript. Data analysed by two-way ANOVA and *t*-tests. n.s. = not statistically significant, * $P \leq 0.05$, ** $P \leq 0.01$, *** $P \leq 0.001$. Data are mean ± s.e.m; (A, C) $n = 20$; (E-J) $n = 2 – 3$. Source data are provided in the Source Data file.

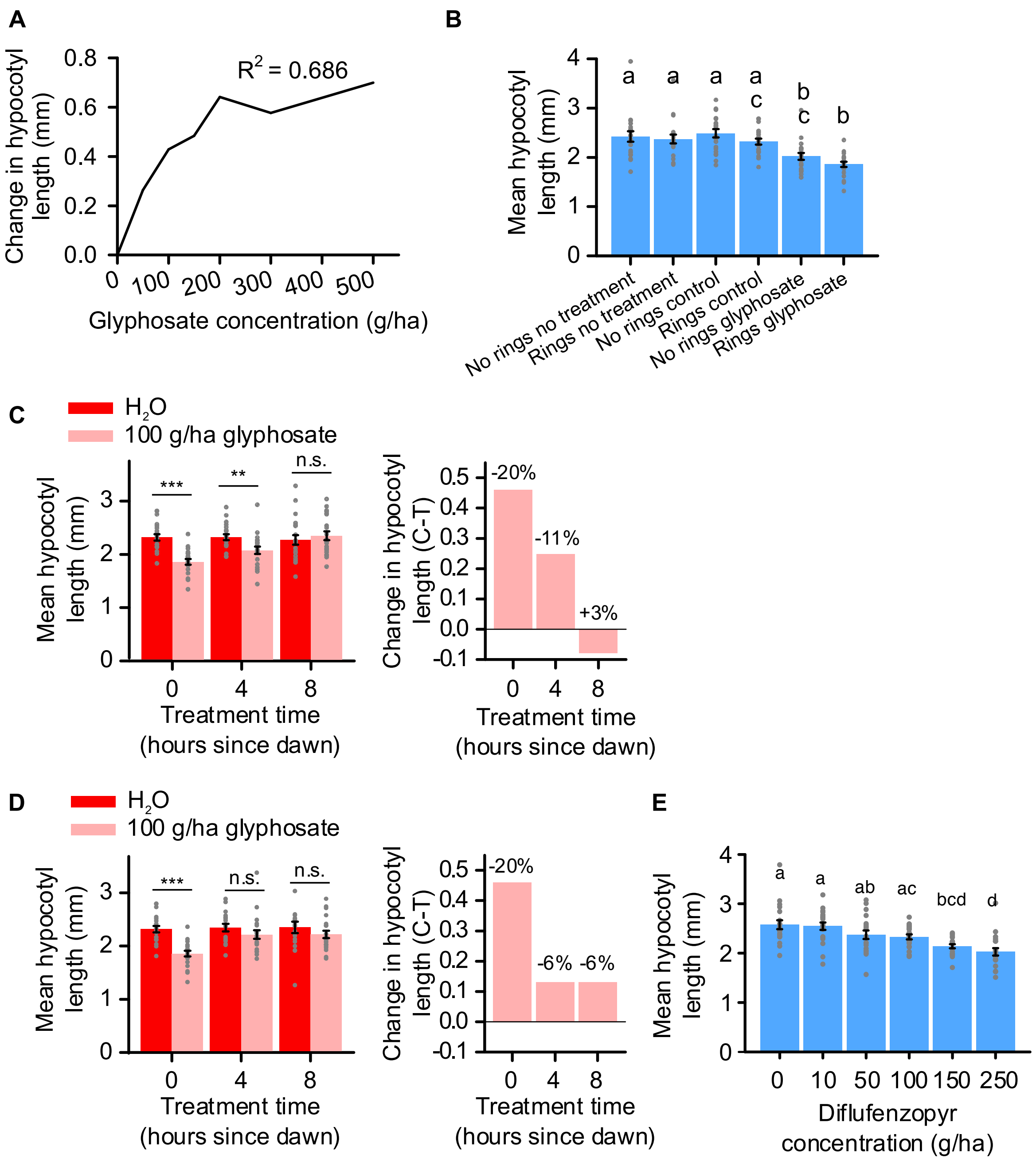

Supplementary Figure 8. Optimization of experimental conditions. (A) Decrease in hypocotyl length caused by range of glyphosate concentrations. Glyphosate applications were at dawn on day 3, measurements on day 7 after germination. (B) Verification that plastic rings used to guide chemical application did not alter hypocotyl length. (C, D) Confirmation that dependency of hypocotyl length upon time of glyphosate application was not caused by measurement of all hypocotyls at the same time; in (C) all hypocotyls were measured at dawn, 4 days after glyphosate treatment at times specified, whereas in (D)

hypocotyls were measured at the time corresponding to exactly 4 days after the glyphosate treatment (e.g. seedlings treated 8 h after dawn were subsequently measured 8 h after dawn, 4 days later). (E) Mean hypocotyl length after a range of diflufenzopyr treatments at dawn. Significance determined by (B, E) one-way ANOVA and post-hoc Tukey analysis and (C, D) two-way ANOVA and t-tests. n.s. = not statistically significant, * $P \leq 0.05$, ** $P \leq 0.01$, *** $P \leq 0.001$. Data are mean ± s.e.m; *n* = 18-20. Source data are provided in the Source Data file.

Supplementary Table 1. Primers used for qRT-PCR experiments.

Primer	Sequence	Reference
PP2AA3 (forward)	TAACGTGGCCAAAATGATGC	[1]
PP2AA3 (reverse)	GTTCTCCACAACCGCTTGGT	[1]
YUC9 (forward)	GTCCCATTCGTTGTGGTCG	[2]
YUC9 (reverse)	TTGCCACAGTGACGCTATGC	[2]
IAA29 (forward)	ATCACCATCATTGCCCGTAT	[2]
IAA29 (reverese)	ATTGCCACACCATCCATCTT	[2]
EXPA8 (forward)	CCGAAGAGTACCATGTATGAAG	[3]
EXPA8 (reverse)	GAGATCAGAACGAGGTTGAAG	[3]
MC1 (forward)	TGGTACCGTTCTGGATTTAC	This paper
MC1 (reverse)	GATGATCCTCCCACACATAC	This paper
DAD1 (forward)	AGGAATTCAAGGATTTAGCAC	This paper
DAD1 (reverse)	CTATCCGAGGAAGTTGATGAT	This paper

Supplementary References

1 Czechowski, T., Stitt, M., Altmann, T., Udvardi, M. K. & Scheible, W.-R. Genome-wide identification and testing of superior reference genes for transcript normalization in Arabidopsis. *Plant Physiology* **139**, 5 (2005).

2 Hayes, S., Velanis, C. N., Jenkins, G. I. & Franklin, K. A. UV-B detected by the UVR8 photoreceptor antagonizes auxin signaling and plant shade avoidance. *Proceedings of the National Academy of Sciences* **111**, 11894 (2014).

3 Simon, N. M. L. *et al.* The energy-signaling hub SnRK1 is important for sucrose-induced hypocotyl elongation. *Plant Physiology* **176**, 1299 (2018).
