# Supplementary material for: Plant circadian rhythms regulate the effectiveness of a glyphosate-based herbicide
Source: Nat Commun. 2019 Aug 16;10:3704. doi: 10.1038/s41467-019-11709-5 (PMC6697731; doi:10.1038/s41467-019-11709-5)
Supplement: Supplementary file 2 — Description of Additional Supplementary Files [file 41467_2019_11709_MOESM2_ESM.pdf]

Description of Additional Supplementary Files

File Name: Supplementary Data 1
Description: Transcripts that are both glyphosate-responsive and circadian or diel-regulated. Genes involved in auxin biosynthesis or signalling are highlighted. List of GO terms related to auxin that were used in a comparison between those and glyphosate and light-dark/circadian regulated lists. Gene names from TAIR genome annotations v11.

File Name: Supplementary Data 2
Description: Statistical analysis of data from all experiments. Sample sizes, statistical tests used and analysis output including p values are provided for all experiments. Each spreadsheet tab contains the analysis for one figure.
