# Supplementary material for: Plant circadian rhythms regulate the effectiveness of a glyphosate-based herbicide
Source: Nat Commun. 2019 Aug 16;10:3704. doi: 10.1038/s41467-019-11709-5 (PMC6697731; doi:10.1038/s41467-019-11709-5)
Supplement: Supplementary file 5 — Reporting Summary [file 41467_2019_11709_MOESM5_ESM.pdf]

nature research

Corresponding author(s): Antony N. Dodd

Reporting Summary

Nature Research wishes to improve the reproducibility of the work that we publish. This form provides structure for consistency and transparency in reporting. For further information on Nature Research policies, see Authors & Referees and the Editorial Policy Checklist.

Statistical parameters

When statistical analyses are reported, confirm that the following items are present in the relevant location (e.g. figure legend, table legend, main text, or Methods section).

n/a	Confirmed	
☐	☒	The exact sample size (n) for each experimental group/condition, given as a discrete number and unit of measurement
☐	☒	An indication of whether measurements were taken from distinct samples or whether the same sample was measured repeatedly
☐	☒	The statistical test(s) used AND whether they are one- or two-sided<br>*Only common tests should be described solely by name; describe more complex techniques in the Methods section.*
☐	☒	A description of all covariates tested
☐	☒	A description of any assumptions or corrections, such as tests of normality and adjustment for multiple comparisons
☐	☒	A full description of the statistics including central tendency (e.g. means) or other basic estimates (e.g. regression coefficient) AND variation (e.g. standard deviation) or associated estimates of uncertainty (e.g. confidence intervals)
☐	☒	For null hypothesis testing, the test statistic (e.g. F, t, r) with confidence intervals, effect sizes, degrees of freedom and P value noted<br>*Give P values as exact values whenever suitable.*
☒	☐	For Bayesian analysis, information on the choice of priors and Markov chain Monte Carlo settings
☒	☐	For hierarchical and complex designs, identification of the appropriate level for tests and full reporting of outcomes
☒	☐	Estimates of effect sizes (e.g. Cohen's d, Pearson's r), indicating how they were calculated
☐	☒	Clearly defined error bars<br>*State explicitly what error bars represent (e.g. SD, SE, CI)*

Our web collection on statistics for biologists may be useful.

Software and code

Policy information about availability of computer code

Data collection	Hypocotyl lengths were measured from images analyzed using ImageJ 1.46r. Luciferase bioluminescence was measured using Photek Image32 software. qRT-PCR data were collected with Agilent MxPro and LinRegPCR (v2018.0) software.
Data analysis	Statistical analysis performed using Sigmaplot 13.0, except for hypergeometric tests that were performed in Excel. Analysis of rhythmic features within luciferase bioluminescence timecourse imaging was performed using Biological Rhythms Analysis Software System (BRASS) (biodare2.ed.ac.uk).

For manuscripts utilizing custom algorithms or software that are central to the research but not yet described in published literature, software must be made available to editors/reviewers upon request. We strongly encourage code deposition in a community repository (e.g. GitHub). See the Nature Research guidelines for submitting code & software for further information.

April 2018

Data

Policy information about availability of data

All manuscripts must include a data availability statement. This statement should provide the following information, where applicable:

- Accession codes, unique identifiers, or web links for publicly available datasets
- A list of figures that have associated raw data
- A description of any restrictions on data availability

All data points are shown within the figures within the paper. Data spreadsheets available upon request.

Field-specific reporting

Please select the best fit for your research. If you are not sure, read the appropriate sections before making your selection.

[x] Life sciences   [ ] Behavioural & social sciences   [ ] Ecological, evolutionary & environmental sciences

For a reference copy of the document with all sections, see nature.com/authors/policies/ReportingSummary-flat.pdf

Life sciences study design

All studies must disclose on these points even when the disclosure is negative.

Sample size	No sample size calculations were performed. Sample size was selected and determined to be adequate based on the consistency and measurability of differences within the data, and informed by other studies using identical methods (e.g. Simon et al. Plant Physiology 2018; Noordally et al. Science 2013).
Data exclusions	No data were excluded from experiments that completed successfully. Experiments that had obviously failed (e.g. contamination on petri dishes and RNA samples that had degraded) were discarded
Replication	All experiments were repeated completely independently at least two times to verify the findings, in addition to the replication performed within each experiment.
Randomization	Petri dish location within growth chambers was randomized to control for small local variations in environmental conditions. For all experiments, plant material was sampled from at least two petri dishes (each harbouring multiple replicate seedlings) to control for variation in the petri dish microenvironment.
Blinding	Blinding was not possible because experimenters had knowledge of the time of day that treatments were applied, and the phenotypes arising from herbicide treatments and circadian arrhythmic genotypes were visible and obvious after experimentation.

Reporting for specific materials, systems and methods

Materials & experimental systems

n/a	Involved in the study
[x]	[ ] Unique biological materials
[x]	[ ] Antibodies
[x]	[ ] Eukaryotic cell lines
[x]	[ ] Palaeontology
[x]	[ ] Animals and other organisms
[x]	[ ] Human research participants

Methods

n/a	Involved in the study
[x]	[ ] ChIP-seq
[x]	[ ] Flow cytometry
[x]	[ ] MRI-based neuroimaging
